# Commentary: The causal role of α-oscillations in feature binding

**DOI:** 10.3389/fnins.2020.00753

**Published:** 2020-08-19

**Authors:** Max A. Crayen, Pinar Yurt, Stefan Treue, Moein Esghaei

**Affiliations:** ^1^Cognitive Neuroscience Laboratory, German Primate Center - Leibniz Institute for Primate Research, Göttingen, Germany; ^2^International Max Planck Research School for Neurosciences, Göttingen, Germany; ^3^Royan Institute for Steam Cell Biology and Technology, ACECR, Tehran, Iran

**Keywords:** binding problem, neural oscillations, alpha oscillations, tACS (transcranial alternating current stimulation), visual attention, EEG - Electroencephalogram, feature binding, visual consciousness

The “binding problem,” the perceptual coupling of visual features that are processed by separate areas of the visual cortex into a joint perceptual object, remains a vexing issue when attempting to link neural activity and perceptual experience. One experimental approach, taken by Wu et al., is to study “misbinding,” i.e., when two given visual feature dimensions (e.g., the color and motion direction) of the *same* object may be perceived as belonging to *different* objects. In this study, the authors designed a visual stimulus with two overlapping random dot patterns, differing in their color (red vs. green) and motion direction (upward vs. downward) and with their left and right panels combining the color and motion inversely compared to the central panel (Wu et al., 2004). They observed that human subjects often perceive the peripheral panels' color/motion combinations the same as the central one (active binding), as opposed to perceiving their physical color/motion combination (physical binding). For instance, when the central upward-moving dots are colored red, subjects perceive the peripheral upward-moving dots also as red, even though the red dots in those panels move downward. In this context, Zhang et al. recently investigated the role of neural oscillations within the alpha frequency band (7–14 Hz) in the perceptual binding of color and motion direction in humans (Zhang et al., 2019). They documented that the power of alpha oscillations decreases when the color and motion direction of the peripheral random dot patterns are actively bound, compared to when they are physically bound. Using transcranial alternating current stimulation (tACS) to modulate the alpha frequency components, they observed changes in the active binding state duration, similarly suggesting that these oscillations have a causal role in visual feature binding. This link between the power of alpha oscillations and perceptual binding is a novel finding; however, the potential influence of attention on this link is yet to be explored.

There are a multitude of mechanistic roles that are associated with alpha oscillations and low frequencies (<20 Hz) in general. These oscillatory activities have been proposed to act as a metronome for processing audio-visual stimuli (Cecere et al., 2015) and are known to control information flow dynamically (Jensen et al., 2014). They are also known to determine the resolution of conscious perception (Wutz et al., 2018). Here we suggest that the role of spatial visual attention needs to be considered when interpreting the link between alpha oscillations and perceptual binding. Particularly, the possible role of attention seemed to be underestimated when evaluating the connection between feature binding and alpha oscillatory activity because some of Zhang et al.'s findings could also be explained by different spontaneous or tACS-induced attentional levels.

The observed suppression of alpha power in active binding, compared to physical binding, is reminiscent of the well-known suppression of low-frequency oscillations by spatial attention (Fries et al., 2001; Khayat et al., 2010; Esghaei and Daliri, 2014); i.e., a higher level of spatial attention allows a more efficient active binding. This is particularly consistent with widely observed differential up and down regulation of neuronal gain by attention (Schwedhelm et al., 2016; Yao et al., 2016; Malek et al., 2017; Kozyrev et al., 2019; Mehrpour et al., 2020). Nevertheless, Zhang et al. hypothesize that it is also the frequency rather than only the power of alpha oscillations that is crucial for binding. Based on previous work, showing that tACS stimulation at a given frequency induces an alpha peak frequency at the stimulation frequency (Minami and Amano, 2017), by injecting alternating currents at different frequencies, Zhang et al. aimed to induce different alpha frequencies, reporting that “delivering tACS at different temporal frequencies in the α-band changed subjects' perceptual switch rate.” However, it is quite likely that at different frequencies these stimulations produce different alpha power modulations. Vossen et al. reported that tACS stimulation at different alpha frequencies does not induce a peak at the stimulation frequency but rather creates a peak at another frequency close to the individual alpha frequency (IAF) of a subject (Vossen et al., 2015). Furthermore, they found that the alpha power enhancement was the strongest when tACS was applied at frequencies lower than the IAF, and even stimulation at frequencies higher than the IAF caused a negative influence on alpha power. This suggests that a stimulation at frequencies *above* the IAF induces a weaker alpha power, whereas stimulations at frequencies *below* the IAF cause stronger alpha powers. Therefore, in light of the previous studies showing that a higher level of attention is linked to a faster as well as a more stable perceptual sampling of the environment (Esghaei et al., 2015; Re et al., 2019), one could assume that the increase in perceptual switch rate at stimulations of higher frequencies may be due to an amplification of attention by suppressing alpha. It is noteworthy that in another study there was no observation of a differential power modulation at different stimulation frequencies (Minami and Amano, 2017). Therefore, we propose that the mechanism underlying the frequency-dependent effect of tACS on the perceptional switch rate could be better understood by comparing the power spectrums of the EEG data under the different stimulation frequencies.

Zhang et al. also observed that subjects with a higher power of alpha had a shorter active binding perception and, further, those with a higher frequency of alpha have a faster perceptual switch rate (shorter active binding epochs). Whether these two findings could be attributed to attention levels should be studied in the future by examining the potential inter-individual correlation between alpha frequency and power and whether subjects with a weaker alpha power allocate higher levels of attention.

Furthermore, changing levels of attention are associated with changes in the power of high frequency oscillations (>30 Hz, Gamma frequency band) (Fries et al., 2001; Jensen et al., 2007; Khamechian et al., 2019). Zhang et al. argue against the possibility of a difference in the attentional strength between the two binding conditions (active vs. physical binding) based on the observation of the power of oscillations within the range of 30–60 Hz, reporting that there was no significant difference in gamma power between the two conditions. However, the authors ignored important attention-relevant gamma bands, such as 80 Hz in humans (Jensen et al., 2007—Figure 1) and 200 Hz in monkeys (Khamechian et al., 2019). It also remains unclear if there is indeed no power difference between the two conditions when looking into smaller sub-bands, an analysis recommended by Jensen et al. (2007). Given the visually apparent difference between the gamma phase synchronization of the two binding conditions within the sub-band ~38–45 Hz (Zhang et al., 2019—Figure S1A), a reanalysis of their data in the gamma and higher ranges could help to clarify if there is any attentional level difference between the binding conditions, in addition to the binding state differences.

In summary, Zhang et al. documented a novel association between alpha frequencies and perceptual feature binding. We propose a more central role of attention as a causal factor for changes in alpha power and frequency of alternations between binding states. Zhang et al. showed in a previous study with controlled levels of attention that physical and active binding may happen at equal attention levels (Zhang et al., 2014); however, it is unclear how changes of attention level may modulate the timing/frequency of these perceptual alternations by affecting alpha power/frequency. Future electrophysiological experiments should employ a parallel attention-demanding task to control the level of attention across the feature-binding task, similar to what Zhang et al. did previously. This may help decipher if alpha power may control feature binding in independence from attention.

## Author Contributions

MC, PY, ST, and ME conceived the ideas. MC and ME wrote the manuscript. ST critically revised the manuscript. All authors approved the final version of the manuscript.

## Conflict of Interest

The authors declare that the research was conducted in the absence of any commercial or financial relationships that could be construed as a potential conflict of interest.
